# New Data on Human Macrophages Polarization by *Hymenolepis diminuta* Tapeworm—An *In Vitro* Study

**DOI:** 10.3389/fimmu.2017.00148

**Published:** 2017-02-20

**Authors:** Anna Zawistowska-Deniziak, Katarzyna Basałaj, Barbara Strojny, Daniel Młocicki

**Affiliations:** ^1^Witold Stefański Institute of Parasitology, Polish Academy of Sciences, Warsaw, Poland; ^2^Division of Nanobiotechnology, Faculty of Animal Sciences, Department of Animal Feeding and Biotechnology, Warsaw University of Life Sciences, Warsaw, Poland; ^3^Department of General Biology and Parasitology, Medical University of Warsaw, Warsaw, Poland

**Keywords:** human, macrophages, Cestoda, *Hymenolepis diminuta*, immunomodulation, immunology, host–parasite interactions

## Abstract

Helminths and their products can suppress the host immune response to escape host defense mechanisms and establish chronic infections. Current studies indicate that macrophages play a key role in the immune response to pathogen invasion. They can be polarized into two distinct phenotypes: M1 and M2. The present paper examines the impact of the adult *Hymenolepis diminuta* (HD) tapeworm and its excretory/secretory products (ESP) on THP-1 macrophages. Monocytes were differentiated into macrophages and cultured with a living parasite or its ESP. Our findings indicate that HD and ESP have a considerable impact on human THP-1 macrophages. Macrophages treated with parasite ESP (with or without LPS) demonstrated reduced expression of cytokines (i.e., IL-1α, TNFα, TGFβ, IL-10) and chemokines (i.e., IL-8, MIP-1α, RANTES, and IL-1ra), while s-ICAM and CxCL10 expression rose after ESP stimulation. In addition, inflammatory factor expression rose significantly when macrophages were exposed to living parasites. Regarding induced and repressed pathways, significant differences were found between HD and ESP concerning their influence on the phosphorylation of ERK1/2, STAT2, STAT3, AMPKα1, Akt 1/2/3 S473, Hsp60, and Hck. The superior immunosuppressive properties of ESP compared to HD were demonstrated with lower levels of IL-1β, TNF-α, IL-6, IL-23, and IL-12p70 following stimulation. The presence of HD and its ESP were found to stimulate mixed M1/M2 macrophage phenotypes. Our findings indicate new molecular mechanisms involved in the response of human macrophages to tapeworm infection, this could be a valuable tool in understanding the mechanisms underlying the processes of immune regulation during cestodiasis.

## Introduction

Macrophages are versatile cells that play crucial roles in the complex process of the immune response to pathogen invasion. As macrophages are key modulator and effector cells in the immune response, their activation influences and responds to other arms of the immune system. It is generally considered that macrophages represent a spectrum of activated phenotypes rather than stable subpopulations (1–3). Typically, macrophages can be polarized into two distinct phenotypes: M1—classically activated macrophages induced by T helper 1 (Th1) cytokines, and M2—alternatively activated macrophages classified as M2a, M2b, M2c, and M2d induced by Th2 cytokines (1–3). Regulation of macrophage function and activity is essential to balance tissue homeostasis, forcing or solving inflammation in most disease processes. The inflammatory or anti-inflammatory activities of macrophages are shaped in a tissue- and signal-specific manner, enabling macrophages to induce various activation patterns and develop specific functional programs (4, 5).

Helminths are known to have coevolved with their hosts for millennia, and the principal goal of the adult parasite is, arguably, not to kill the host but to survive as long as possible by generating a state of tolerance. This state of affairs is beneficial for the parasitic organism as the host provides nutrition, protection, and stable conditions for growth. Therefore, dendritic cells and macrophages are among the first cells to be encountered by the parasite, which, by expressing certain molecules, has developed complex mechanisms to escape and modulate host immunity. One of these mechanisms exploits the impact of parasite surface proteins or their excretory/secretory products (ESP) on macrophage polarization type.

Both the M1 and M2 phenotypes are involved in the parasite invasion to various extents depending on parasite type and life cycle. In general, macrophages undergo a dynamic switch toward the M2 phenotype. In the case of *Taenia crassiceps*, while the M1 phenotype was observed during the early stage of infestation, the M2 phenotype later become dominant as the infection progressed, with a decreased parasite burden (6).

The influence of helminth-derived products on immune systems has been extensively studied (7–14), particularly with regard to the value of helminth products as antigens displaying immunomodulatory properties. The immunomodulatory properties of helminth-derived molecules have been screened for *Hymenolepis diminuta* (HD) (9, 15–18); these data show that HD may represent a source of anti-inflammatory and immunomodulatory molecules.

Experiments performed on animal models of human autoimmune diseases have shown that parasites can be beneficial and may have therapeutic potential in treatment of autoimmune disorders (10–12, 19, 20). Despite increasing knowledge of the influence of parasites on the host immune system, numerous mechanisms involved in this process seem to be unknown. Therefore, the ultimate goal of our study was to find new molecular pathways present in macrophages exposed to adult tapeworms. To achieve our goal, we used adult HD, commonly known as rat tapeworm, which is able to establish a chronic infection in the small intestine of the host with minimal influence on the intestinal tissue. *Hymenolepis* does not cause serious damage and influences the rat host immune system at the molecular level, producing proteins with antigenic properties (9, 15–18, 21–24). In addition, the ability to infect both animals and man makes this parasite a valuable model to study the influence of the parasite on its host, and since the regulatory mechanisms of rats and humans are comparable, host–parasite interactions such as immunomodulation can also be examined.

In light of the immunomodulatory properties of parasites and the importance of macrophages in numerous serious diseases, there is a need for more comprehensive research regarding the interactions and role of macrophages during parasite infections. Therefore, the aim of the present study was to characterize the polarization type of human THP-1 macrophages following stimulation with living HD and its ESP. We chose the THP-1 human leukemia monocytic cell line as it has been extensively used to study monocyte/macrophage functions, mechanisms, and signaling pathways. As our analysis was aimed at screening for changes and looking for new possible pathways induced by the parasite, we decided to use a cell line to select the most interesting factors, which will be carefully studied in the future using primary cells. The results obtained using this model are comparable to primary human PBMC–monocytes as indicated by a number of publications that have compared responses of both cell types and in most cases showed relatively similar response patterns (25–27). Certainly, THP-1 macrophages represent an alternative to PBMC–macrophages for screening purposes, when looking for new mechanisms and a homogeneous genetic background is wanted (28). This is especially the case when the availability of PBMC-derived macrophages is often limited, and insufficient quantities are available to perform broad analyses. Due to either financial or ethical constraints linked to animal and human *in vivo* studies, *ex vivo* or *in vitro* experiments become more relevant in initial screening research. Additionally, commercially available proteome arrays allow for comprehensive analysis where all experiments are performed in the same conditions. The dozens of analyzed factors allow for complex assessment and predictions regarding unstudied mechanisms. The THP-1 cell line has become a commonly used model to assess the modulation of macrophage activities and represents a competent *in vitro* model for estimation of the immunomodulatory properties of parasite proteins. For example, previous studies have utilized THP-1 cells to examine human monocyte/macrophage stimulation in response to parasite proteins (17, 29–31). A key novel aspect of the present study is that it is the first to comprehensively characterize the impact of the living parasite and its ESP on human THP-1 macrophages. The obtained results highlight the significance of a number of factors concerning the immunomodulatory properties of parasite proteins that have yet to be studied.

## Animals and Methods

### Experimental Animals

Male Lewis rats aged about 3 months at the beginning of the experiment, to be used as experimental hosts, were kept in plastic cages in the animal house facilities of the Institute of Parasitology PAS. They had continuous access to food and water, and natural photoperiod conditions were provided.

### Ethics Statement

All experimental procedures used in the present study had been preapproved by the third Local Ethical Committee for Scientific Experiments on Animals in Warsaw, Poland (resolution no. 51/2012, 30th of May 2012).

### Cultivation of HD and Collection of ES Products

The HD strain was kept in the Institute of Parasitology PAS (strain WMS). Six-week-old cysticercoids reared in *Tribolium castaneum* beetles were fed in doses of 8–10 to 3-month-old rats (15 male rats). After 6 weeks, coproscopic examination of the rat feces was performed to ascertain the presence of adult parasites. To collect the adult parasites, the rats were euthanized with Thiopental anesthesia (Biochemie GmbH, Austria), administered in 100 mg/kg body weight (b.w.) intraperitoneally (i.p.).

Adult HD were obtained from the small intestine of infected rats and washed few times in PBS at room temperature to remove intestinal debris. The worms were incubated at 37°C in RPMI 1640 culture media containing penicillin and streptomycin (Sigma) for 10 h, with the media changed every 2 h. The harvested media containing ESP were pooled and centrifuged at 5,000 rpm for 15 min and directly placed in an Amicon^®^ Ultra Centrifugal Filters Ultracel-3K (Millipore) to concentrate them. Protein concertation was determined with Bradford protein assay. Prepared ESP samples were stored at −80°C until used.

### Cell Culture and Stimulation

The THP-1 human monocyte cell line was purchased from the American Type Culture Collection. Cells were maintained in culture medium (RPMI 1640 supplemented with 10% fetal bovine serum, 2 mM glutamine, 100 U/ml penicillin, 100 µg/ml streptomycin) at 37°C in a humidified atmosphere of 5% CO_2_. The cells were seeded into six-well plates at a concentration of 1 × 10^6^/ml in a whole volume of 4.8 ml/well. The cells were differentiated into macrophages by the addition of 100 ng/ml phorbol 12-myristate 13-acetate (PMA) for 72 h. After differentiation, the cells were washed twice with fresh media w/o PMA and stimulated with ES or whole parasite. For whole parasite stimulation, the cells were maintained in Nunc polystyrene (PS) EasYFlask™ 25 cm^2^ (Thermo Scientific) flasks at the same cell concentration and density per square centimeter. In the case of cells stimulated with parasite antigens and LPS, the cells were first treated with LPS (100 ng/ml), and parasite (one 10-cm worm/10 × 10^6^ cells) or antigens (5 µg/ml) were added after 1 h. After 24 h, the stimulation culture media was collected and cells were washed with sterile PBS. Cells for phosphokinase analysis were lysed with lysis buffer from Proteome Profiler kit (R&D), cells for RNA isolation were directly treated with fenozol supplied with Total RNA kit (A&A Biotechnology) and stored at −80°C until use.

### cDNA Synthesis and Real Time PCR Analysis

Total RNAs were isolated from the same number of cells stimulated with ES products or whole parasite according to the kit manufacturer’s instructions. First-strand cDNAs were synthesized from 0.7 µg of total isolated RNA using a Maxima™ First Strand cDNA Synthesis Kit for RT-qPCR (Thermo Scientific). qPCR were performed by use of Luminaris Color HiGreen High ROX qPCR master Mix (Thermo Scientific). Reactions were conducted in 10 µl of total volume in StepOne Real-Time PCR System, Applied Biosystems.

Gene-specific primers, presented in Table 1, were intron-spanning and purchased from Sigma. Primer sequences were designed or taken from Jaguin et al. (32). Two reference genes were used (β-actin, RPL37A) (33). All primer pairs were designed to have a melting point of about 64°C. Reaction runs included 2 min at 50°C and 10 min at 95°C followed by 40 cycles of a two-step PCR consisting of a denaturing phase at 95°C for 15 s and a combined annealing and extension phase at 72°C for 30 s. The *C*_T_ value of β-actin and RPL37A was subtracted from that of the gene of interest to obtain a Δ*C*_T_ value. The Δ*C*_T_ value of the least abundant sample at all time points for each gene was subtracted from the Δ*C*_T_ value of each sample to obtain a ΔΔ*C*_T_ value. The gene expression level relative to the calibrator was expressed as $2^{-\text{ΔΔ}C_{\text{T}}}$ (34).

**Table 1:** **Primers sequences used for Real-Time PCR**.

Gene	Name	Forward primer	Reverse primer	Source
TNFα	Tumor necrosis factor alpha	5′CCCATGTTGTAGCAAACCCT	5′CCCTTGAAGAGGACCTGG	sd
IL-1β	Interleukin-1 beta	5′GGACAAGCTGAGGAAGATGC	5′TCGTTATCCCATGTGTCGAA	sd
IL-8	Interleukin-8	5′CAAACCTTTCCACCCCAAAT	5′CTCTGCACCCAGTTTTCCTT	sd
IL-12 p35	Interleukin-12 p35	5′GATGGCCCTGTGCCTTAGTA	5′TCAAGGGAGGATTTTTGTGG	(32)
IL-10	Interleukin-10	5′CCTGGAGGAGGTGATGCCCCA	5′CCTGCTCCACGGCCTTGCTC	sd
TGFβ	Transforming growth factor beta	5′TGCGCTTGAGATCTTCAAA	5′GGGCTAGTCGCACAGAACT	(32)
CCL1	CC chemokine type 1	5′ATACCAGCTCCATCTGCTCC	5′TGCCTCAGCATTTTTCTGTG	sd
CCL3	CC chemokine type 3 (MIP-1α)	5′ACTTTGAGACGAGCAGCCAGTG	5′TTTCTGGACCCACTCCTCACTG	sd
CCL4	CC chemokine type 4 (MIP-1β)	5′GTAGCTGCCTTCTGCTCTCC	5′ACCACAAAGTTGCGAGGAAG	sd
CCL22	CC chemokine type 22	5′ATTACGTCCGTTACCGTCTG	5′TAGGCTCTTCATTGGCTCAG	(32)
CCR7	C-C chemokine receptor type 7	5′GTGGTGGCTCTCCTTGTCAT	5′TGTGGTGTTGTCTCCGATGT	(32)
CXCL11	CXC chemokine type 11 (I-TAC)	5′CCTGGGGTAAAAGCAGTGAA	5′TGGGATTTAGGCATCGTTGT	(32)
CHI3L-1	Chitinase-3-like protein 1	5′GATAGCCTCCAACACCCAGA	5′AATTCGGCCTTCATTTCCTT	(32)
CD36	Cluster of differentiation 36	5′AGATGCAGCCTCATTTCCAC	5′GCCTTGGATGGAAGAACAAA	(32)
CD54	Cluster of differentiation 54 (sICAM)	5′GGCTGGAGCTGTTTGAGAAC	5′AGGAGTCGTTGCCATAGGTG	sd
IDO1	Indoleamine 2,3-dioxygenase 1	5′GCGCTGTTGGAAATAGCTTC	5′CAGGACGTCAAAGCACTGAA	(32)
IRF3	Interferon regulatory factor 3	5′AAGAAGGGTTGCGTTTAGCA	5′TCCCCAACTCCTGAGTTCAC	(32)
Klf4	Krueppel-like factor 4	5′CCCACACAGGTGAGAAACCT	5′ATGTGTAAGGCGAGGTGGTC	(32)
MRC1	Mannose receptor C type 1	5′GGCGGTGACCTCCACAAGTAT	5′ACGAAGCCATTTGGTAAACG	(32)
NFκB p65	Nuclear factor kappa B p65 (RelA)	5′TCTGCTTCCAGGTGACAGTG	5′ATCTTGAGCTCGGCAGTGTT	(32)
PPARc	Peroxisome proliferator-activated receptor c	5′TTCAGAAATGCCTTGCAGTG	5′CCAACAGCTTCTCCTTCTCG	(32)
MHC I	Major histocompatibility complex 1	5′GCAGTTGAGAGCCTACCTGG	5′CTCATGGTCAGAGATGGGGT	sd
MHC II	Major histocompatibility complex 2	5′AGGCAGCATTGAAGTCAGGT	5′CTGTGCAGATTCAGACCGTG	sd
RPL37A	Ribosomal protein L37a	5′ATTGAAATCAGCCAGCACGC	5′AGGAACCACAGTGCCAGATC	(33)
ACTB	β-actin	5′ATTGCCGACAGGATGCAGAA	5′GCTGATCCACATCTGCTGGAA	(33)

*sd, self-designed primers*.

### Phospho-Kinase Arrays

The phospho-antibody array analysis was performed using the Proteome Profiler Human Phospho-Kinase Array Kit from R&D Systems according to the manufacturer’s instructions. After a 24-h stimulation period, macrophages were lysed with Lysis Buffer 6 (R&D Systems) and agitated for 30 min at 4°C. Cell lysates were clarified by microcentrifugation at 14,000 × *g* for 5 min, and the supernatants were subjected to protein assay using a Pierce ™ BCA Protein Assay Kit (Thermo Scientific). Preblocked nitrocellulose membranes of the Human Phospho-Kinase arrays were incubated with ~400 μg (ES/whole parasite stimulation w/o LPS) or ~240 μg (stimulation with LPS) of cellular extract overnight at 4°C on a rocking platform. The membranes were washed three times with 1× Wash Buffer (R&D Systems) to remove the unbound proteins and were then incubated with a mixture of biotinylated detection antibodies and streptavidin-HRP antibodies. Chemiluminescent detection reagents were applied to detect spot densities. Membranes were exposed to X-ray film for 3, 5, and 10 min. Array images were analyzed using image analysis software Quantity One (Biorad).

### Cytokine Arrays

The collected culture media from cells stimulated with parasite and ES products were subjected to the Proteome Profiler Human Cytokine Array Panel A (R&D Systems) according to the manufacturer’s instructions. Each nitrocellulose membrane contains duplicated spots of 36 different antibodies for anticytokines, chemokines, growth factors, and adhesion proteins. Preblocked nitrocellulose membranes of the Human Cytokine Array were incubated with 1 ml of each culture media and detection antibody cocktail overnight at 4°C on a rocking platform. The membranes were washed three times with 1× Wash Buffer (R&D Systems) to remove unbound proteins. Chemiluminescent detection reagents were applied to detect spot densities. Membranes were exposed to X-ray film for 3, 5, and 10 min. Array images were analyzed using the image analysis software (Quantity One).

### ELISAs

Cytokine (TNF-α, IL-1β, IL-6, IL-12p70, IL-10) concentrations were determined using the commercial ELISA kits OptEIA ™ Set Human (BD Biosciences) and DuoSet ELISA (R&D Systems) for IL-23. Supernatants were stored at −80°C until assayed. Experiments yielding supernatants were performed independently in triplicate. Optical densities were read at the appropriate wavelength on a microplate reader, and measurements were calculated as mean ± SE.

### Statistical Analysis

Δ*C*_T_ values for all genes were normalized to mean *C*_T_ of β-actin and RPL37 reference genes. Δ*C*_T_ values for treated samples and controls (calibrators) were compared by *t*-test for independent samples. Differences at *P* < 0.05 were considered as significant. Analyses were performed using Statgraphics Centurion ver. XV (StatPoint Technologies, Warrenton, VA, USA) (***P < 0.001, **P < 0.01, *P < 0.05). The same *t*-test was used for the analysis of ELISA experiments.

## Results

### HD and Its ESP Impact on THP-1 Macrophage Gene Expression Levels

**Figure 1:**
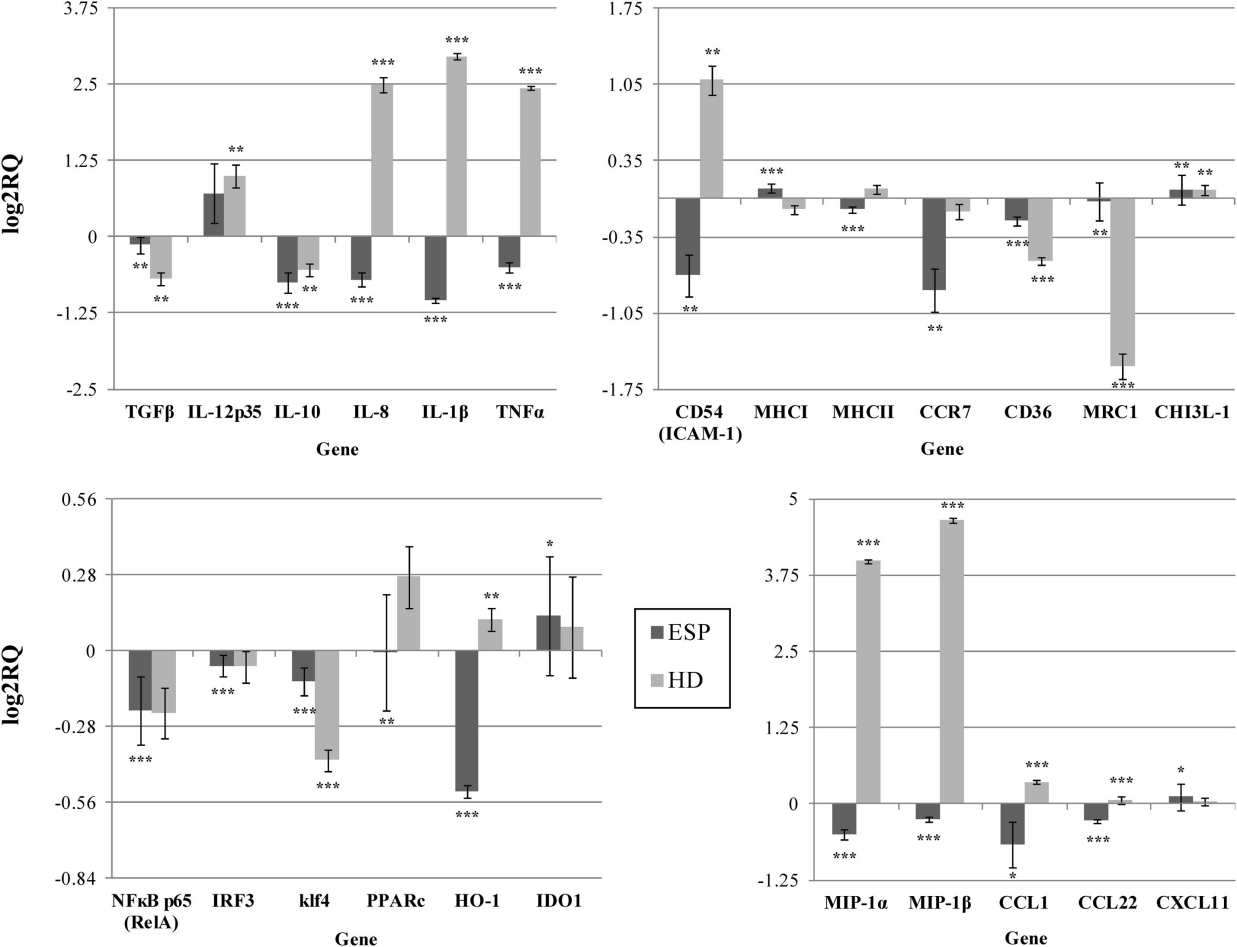
**Gene expression profile without LPS**. Gene expression analysis was determined by qPCR. The results are calculated relative to control, where excretory/secretory products (ESP) and *Hymenolepis diminuta* (HD) had seperated one. Δ*C*_T_ values for all genes were normalized to mean *C*_T_ of β-actin and RPL37 housekeeping genes. Δ*C*_T_ values for treated samples and controls (calibrators) were compared by *t*-test for independent samples. Differences at *P* < 0.05 were considered as significant (***P < 0.001, **P < 0.01, *P < 0.05).

**Figure 2:**
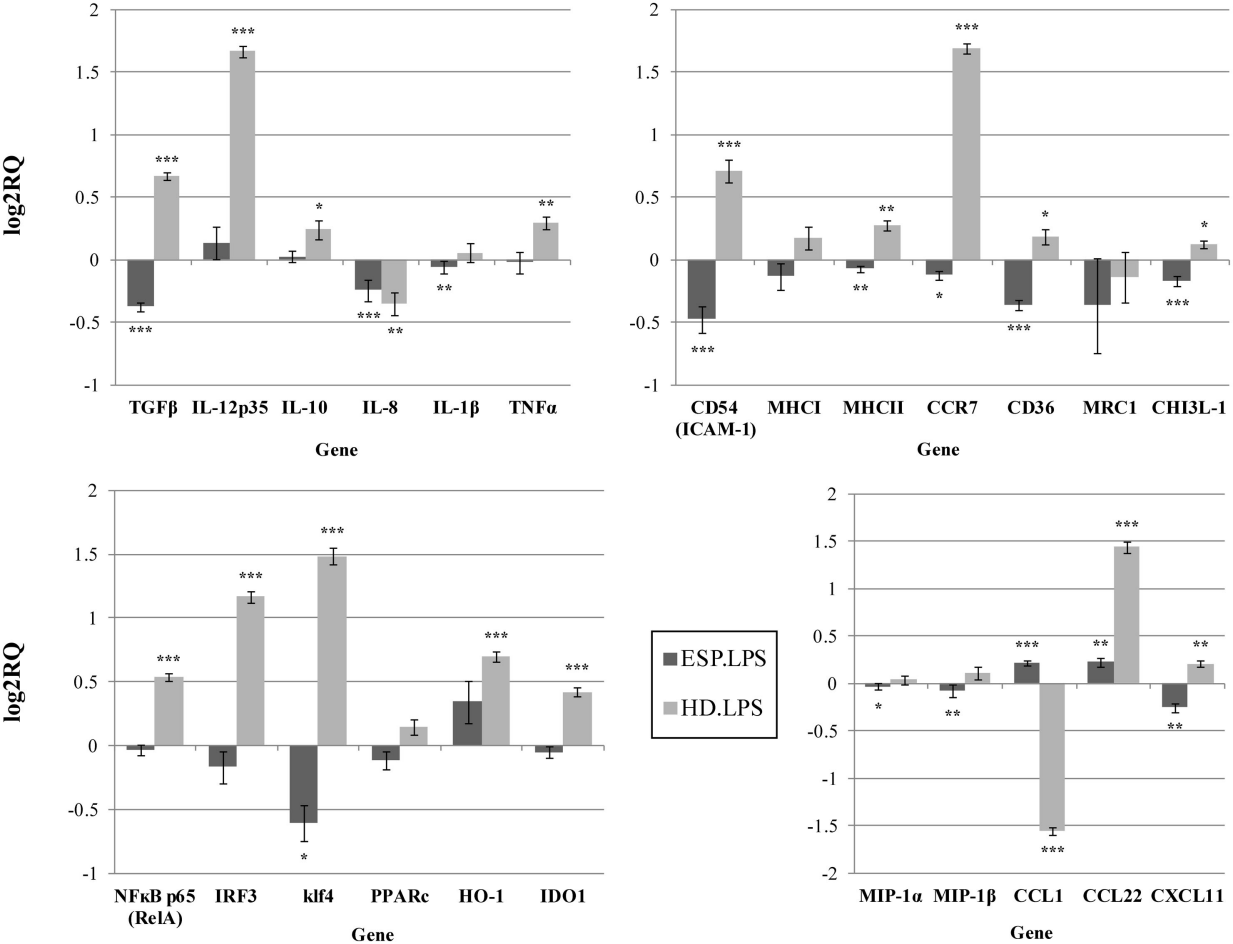
**Gene expression profile with LPS**. Gene expression analysis was determined by qPCR. The results are calculated relative to the LPS control, where excretory/secretory products (ESP) + LPS and *Hymenolepis diminuta* (HD) + LPS had seperated one. Δ*C*_T_ values for all genes were normalized to mean *C*_T_ of β-actin and RPL37 housekeeping genes. Δ*C*_T_ values for treated samples and controls (calibrators) were compared by *t*-test for independent samples. Differences at *P* < 0.05 were considered as significant (***P < 0.001, **P < 0.01, *P < 0.05).

Our findings indicate that HD ESP have a significant inhibitory effect on macrophage-originated inflammatory cytokines and chemokines. In order to evaluate the impact of the HD ESP on macrophage activation, their effect on the expression of pro-inflammatory and anti-inflammatory cytokines and chemokines mRNA was investigated. Stimulation of THP-1 macrophages with ESP, with or without LPS, significantly reduced the expression of *TNF-*α, *IL-1*β, *MIP-1*α, *MIP-1*β, *IL-8*, and *TGF-*β (Figures 1 and 2). The expression of scavenger receptor *CD36*, major histocompatibility complex (*MHC) II, CCR7*, and transcriptions factors *Klf4, IRF3*, and *NF*κ*B p65* were also diminished.

The expression of the *CCL1, CCL22, CXCL11*, and *IL-10* genes differed depending on the addition of LPS to cells stimulated with ESP. *CCL1* and *CCL22* expression was downregulated, but upregulated in the presence of LPS. Additionally, *CHI3L-1, MHC I, HO-1*, and *IDO 1* expression was also dependent on LPS stimulation (Figures 1 and 2). However, treatment with the living parasite triggered a significantly different profile of cytokine expression. The presence of the parasite upregulated the expression of inflammatory cytokines and chemokines such as *TNF-*α, *IL-1*β, *MIP-1*α, *MIP-1*β, and *IL-8* (Figure 1). This increase in expression changed after stimulation with the parasite and LPS (Figure 2): *IL-8* expression was significantly reduced, and anti-inflammatory cytokines such as *TGF-*β and *IL-10* were upregulated. The expression of the scavenger receptor *CD36* and chemokine receptor *CCR7* were enhanced in cells treated with living parasite and LPS, and downregulated in those treated with parasite only. Analogous effects were noted for transcription factors *Klf4, IRF3*, and *NF*κ*B p65*, while *HO-1* levels were comparable in all cells, irrespective of LPS stimulation.

### Cytokine and Chemokine Protein Profile after HD and ESP Stimulation

**Figure 3:**
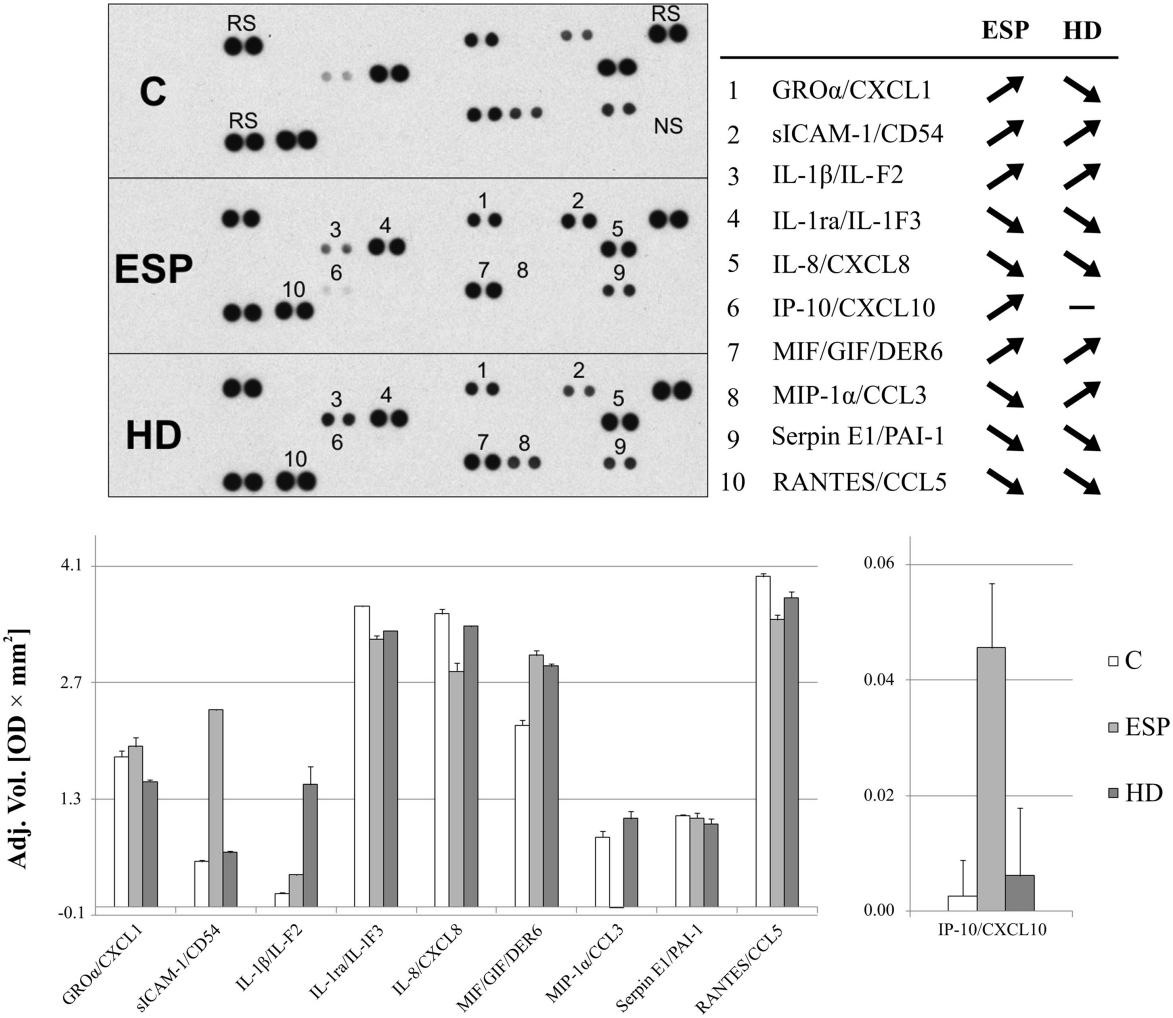
**Cytokine array analysis**. Secreted protein profile in the supernatants of THP-1 macrophages stimulated 24 h with excretory/secretory products (ESP), *Hymenolepis diminuta* (HD), or unstimulated was determined by Protein Profiler Array Panel A. Supernatants of three independent experiments were mixed then subjected for the assay. As a result, we show the adjusted mean volume (OD × mm^2^) ± SD of two repeats on membrane. The average intensity of the pixels in background volume was calculated and subtracted from each pixel in all standard and unknown.

Proteome Profiler cytokine array analysis confirmed inhibited MIP-1α expression in macrophages treated with ESP (ESP/M) and upregulation in macrophages cultured with living HD (HD/M) (Figure 3). The cytokine array analysis indicated that ESP and the live parasite have different effects on CXCL1, sICAM-1, and IL-1β levels in the cell culture medium. Additionally, the arrays reveal variations in the secretion of IL-1ra, MIF, and RANTES: MIF level was strongly induced by both types of stimulation, and IL-8, IL-1ra, SERPIN E1, and RANTES were reduced in comparison to unstimulated cells. While ESP stimulation strongly induced the production of CXCL10, minimal induction was seen in cells treated with the living parasite.

**Figure 4:**
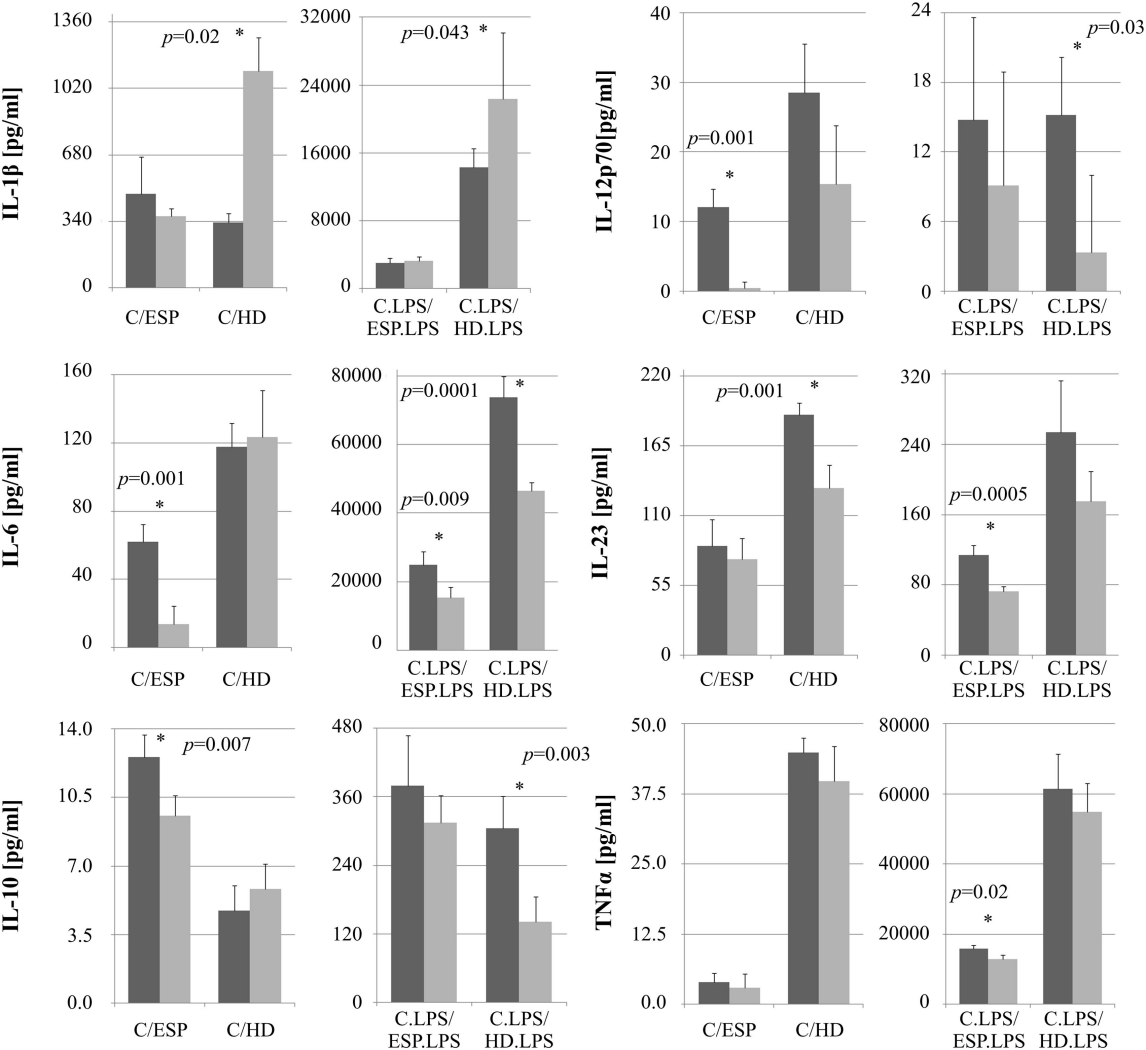
**Cytokine ELISA analysis**. Cytokine production by monocytes THP-1 differentiated to macrophages and stimulated with *Hymenolepis diminuta* (HD) and ES products of this parasite [excretory/secretory products (ESP)]. Cells (1 × 10^6^/ml) were stimulated *in vitro* for 24 h with whole parasite or 5μg/ml ES products (*light gray bar*) or unstimulated (*dark gray*) or in combination with LPS (100 ng/ml). Results are expressed as a mean ± SD of four independent experiments. Statistical analysis was performed by Student’s *t*-test. A value of *P* < 0.05 was considered to be significant.

The levels of selected cytokines were further investigated with ELISA (Figure 4). No significant difference was observed between cells stimulated with ESP or HD, with or without LPS, with regard to TNF-α concentration. While IL-1β secretion rose in macrophages treated with either HD or HD + LPS compared to control cells, no significant changes were observed in those treated with ESP. IL-6 secretion fell in THP-1 macrophages cultured with ESP, ESP + LPS, and HD + LPS, but not in HD. Both the parasite and ESP suppressed the secretion of inflammatory cytokines such as IL-12p70 and IL-23. A similar effect was found for the anti-inflammatory cytokine IL-10, with the exception of the HD cultures, where the IL-10 level found to be higher than controls.

### Changes in Kinase Phosphorylation Profiles in THP-1 Macrophages after ESP and HD Treatment

**Figure 5:**
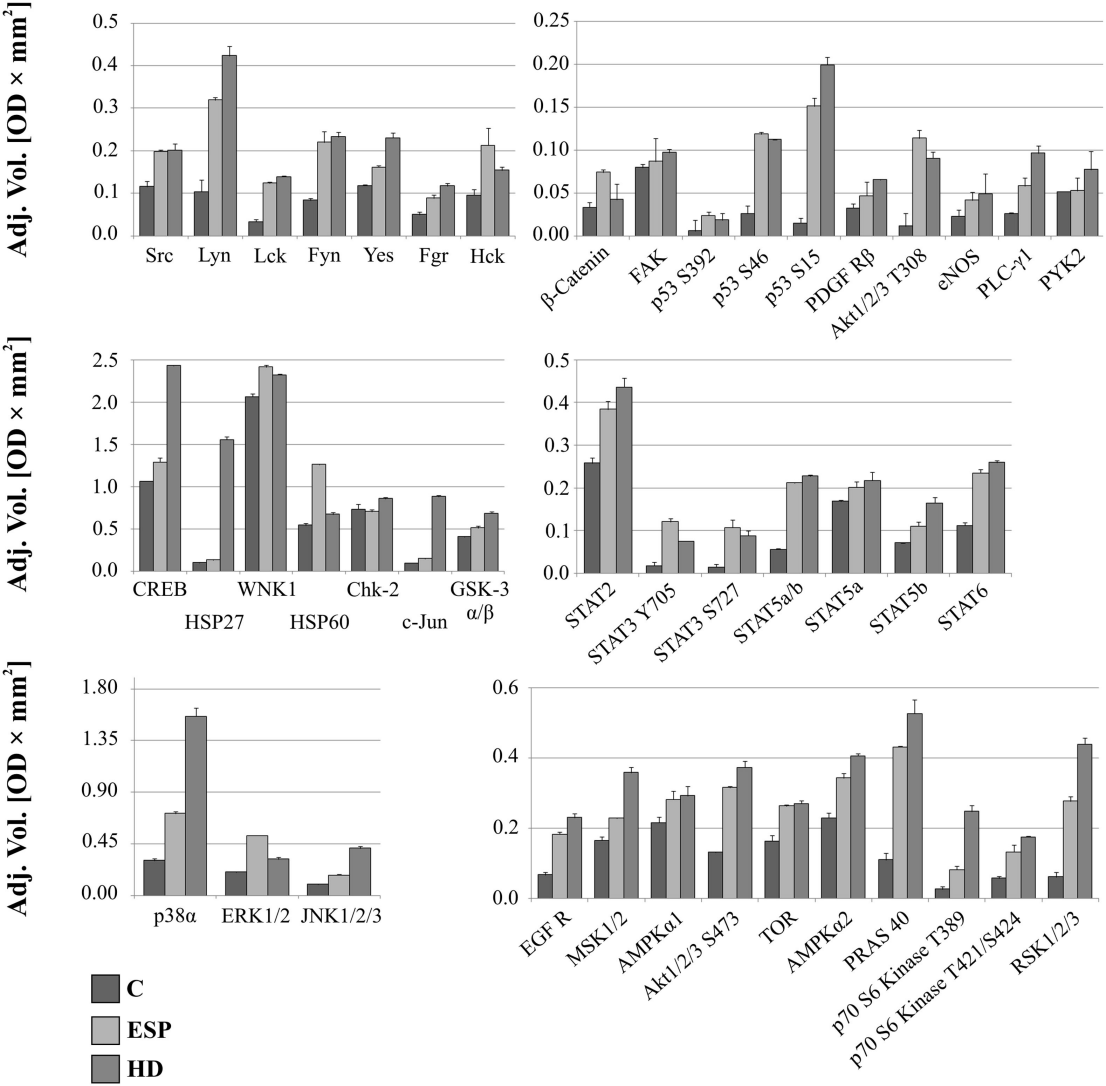
**Phospho-kinase analysis without LPS**. Changes in signaling proteins phosphorylation profile in macrophages stimulated with excretory/secretory products (ESP) and *Hymenolepis diminuta* (HD) was determined by Proteome Profiler Human Phospho-Kinase Array Kit. Stimulated cells were lysed with usage of special kit buffer and frozen in −80°C until use. Protein concentration was checked and the same amout (400 µg) was used for each analysis. Analysis of control and treated sample has to be performed at one time. As a result we show the adjusted mean volume (OD × mm^2^) ± SD of two repeats on membrane. The average intensity of the pixels in background volume was calculated and subtracted from each pixel in all standard and unknown.

The screening analysis of the phosphorylation profiles of selected kinases in cells suggests that both ESP and HD have a similar effect. Only slight changes were observed when phosphorylation levels of selected kinases were increased more for cells stimulated with ESP than HD: p53 (S392, S46 but not for S15), Akt 1/2/3 (T308), β-catenin, STAT3 (Y705, S727), ERK1/2 (T202/Y204, T185/Y187), Hck (Y411), WNK1, and HSP60 (Figure 5; Figure S1 in Supplementary Material).

**Figure 6:**
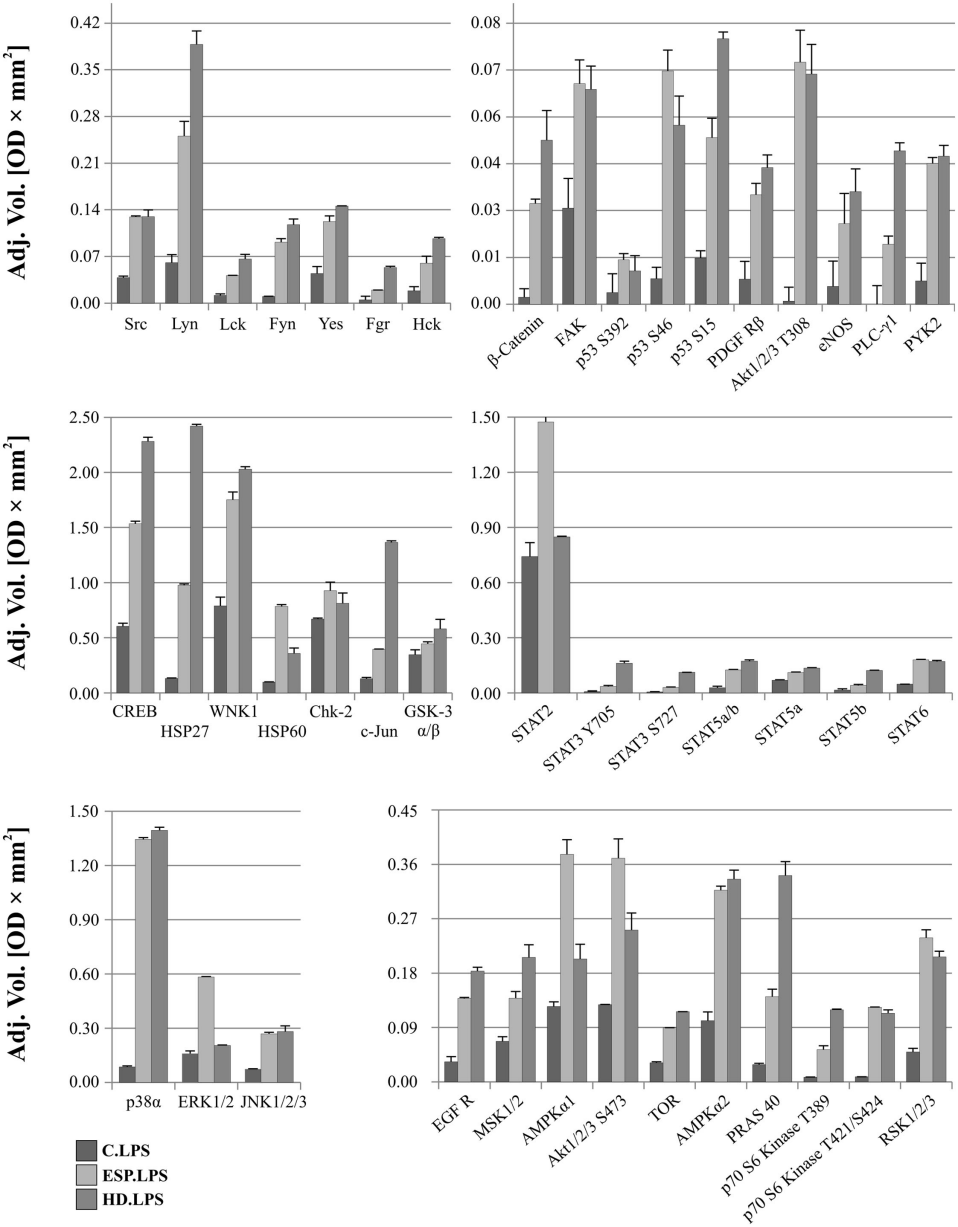
**Phospho-kinase analysis with LPS**. Changes in signaling proteins phosphorylation profile in macrophages stimulated with excretory/secretory products (ESP)/LPS and *Hymenolepis diminuta* (HD)/LPS was determined by Proteome Profiler Human Phospho-Kinase Array Kit. Stimulated cells were lysed with usage of special kit buffer and frozen in −80°C until use. Protein concentration was checked and the same amout (240 µg) was used for each analysis. Analysis of control and treated sample has to be performed at one time. As a result, we show the adjusted mean volume (OD × mm^2^) ± SD of two repeats on membrane. The average intensity of the pixels in background volume was calculated and subtracted from each pixel in all standard and unknown.

After LPS stimulation, the phosphorylation levels of p53, Akt1/2/3 T308, ERK1/2, and HSP60 were similar to those noted for cells without LPS (Figure 6). However, AMPKα1 (T188), Akt1/2/3 (S473), RSK1/2/3 (S380/S386/S377), Chk-2 (T68), p70S6 (T421/S424), STAT2 (Y689), and STAT6 (Y641) had increased phosphorylation in cells treated with ESP and LPS (Figure 6), but not in cells treated with ESP without LPS (Figure 5). The β-catenin, STAT3 (Y705, S727), Hck, and WNK1 in ESP + LPS cells demonstrated reduced levels of phosphorylation compared to HD + LPS cells, whereas the opposite was noted in cell cultures without LPS. HSP27, CREB c-JUN, JNK, and PYK2 demonstrated higher phosphorylation in HD-treated cells than ESP-treated cells, in which phosphorylation was lower or at the same level as control cells.

## Discussion

It is believed that parasitic proteins polarize macrophages principally toward type M2 (35), but the detailed mechanisms underlying this process are as yet unknown. Our results, however, indicate a mixed type of macrophage polarization, which is induced following exposure to HD ESP and the living parasite. Johnston et al. (17) described the general influence of HD antigens on THP-1 macrophages. Similarly to Johnston et al., we also identified anti-inflammatory properties of HD, as we observed inhibition of TNF-α, IL-6, and IL-1β after ESP stimulation. However, in our analyses, stimulation with whole living parasite induced proinflammatory cytokines, whereas Johnston et al. (17) observed no inflammatory stimulation with a high molecular mass extract of HD; there was no IL-1β stimulation, unlike with the HD treatment in our studies. Analysis of ERK1/2 phosphorylation revealed increased levels in ESP and HD, while with *Hd*HMW, there was no effect. Additionally, we also identified inhibition of proinflammatory molecules such as IL-12p70 and IL-23 in ESP and whole parasite treatment, which was not described in the previous reports. These additional results indicate that the anti-inflammatory properties of HD-derived antigens are stronger than was previously known. In relation to previous studies of the influence of the adult tapeworm on macrophages, our results draw attention to several new factors that have not been examined in this respect before. In our studies, we found several markers characteristic for both types of polarization, M1 and M2. This may indicate that parasites evolved mechanisms leading to diminished host reaction to foreign antigens, and this adaptation results in the stimulation of anti-inflammatory pathways in the host organism. Namely, as the host reacts to adult parasite surface antigens, helminths secrete a number of immunomodulatory molecules allowing them to avoid being expelled. This may account for the anti-inflammatory effect of HD ESP. Present data showed distinct cytokine and chemokine expression profiles in the macrophages stimulated with ESP and those with the living HD parasite, some of these results point to new aspects of parasite–host interactions, which are discussed below.

Our results suggest that while ESP, ESP + LPS, and HD + LPS stimulation inhibits the expression of the CXC motif chemokine ligand 8 (*CXCL-8*) gene, the presence of the parasite in the medium enhances the expression level, which is inconsistent with the result of antibody arrays. This might be partially explained by poor protein stability or post-translational mechanisms influencing proteins expression. Some of the factors and mechanisms reflecting the discrepancies in mRNA and protein expression are known and described (36). However, we are unable to establish which mechanism influencing protein abundance was present in our study. The function of IL-8/CXCL8 is to orchestrate the recruitment of neutrophils, basophils, and T-cells, but not monocytes, within inflamed tissues, and is also involved in neutrophil activation (37). The same may be true during the interaction of adult cestodes with host immune cells.

Another chemokine observed by us, which is important for the protective immunity of the host is macrophage inflammatory protein 1 α (MIP-1α/CCL3). The absence of this chemokine greatly impairs the recruitment of monocytes and neutrophils into infected organs. Furthermore, CCL3 induces macrophage activation and the killing of *Escherichia coli, Trypanosoma cruzi*, or *Klebsiella pneumonia* (38, 39). Our results indicate that stimulation of macrophages with HD ESP significantly reduces the level of the MIP-1α protein, as well as its gene expression, although different expression is observed when cells are incubated with the whole parasite. While the mRNA level is highly induced, the protein level reflects this trend in lesser extent, which may be associated with the relatively short half-life of CCL3 protein (40).

Our study showed that stimulation with HD and its ESP decreased the level of the CCL5 (RANTES) chemokine, which shares a common receptor with CCL3 and CCL4, namely, chemokine receptor 5 (CCR5). RANTES is primarily involved in the migration of monocytes, neutrophils, dendritic cells, and T-cells. For instance, microbial challenge with *Toxoplasma gondii* is able to enhance the production of CCL3, CCL4, and CCL5. These chemokines activate CCR5 and signal the production of IL-12 by CD8α dendritic cells to initiate a Th1 response for clearance of the parasite (41). However, M2 macrophages obtained from mice implanted intraperitoneally with the filarial nematode *Brugia malayi* display IL-4-dependent inhibition of the pro-inflammatory chemokines CCL3 and CCL4 (42). During *T. cruzi* infection, CCL5 plays an important protective role in mobilizing B cell populations and is directly able to induce B cell proliferation and IgM secretion (43).

According to the analysis of *CCL1* and *CCL22* gene expression levels in the present study, ESP-stimulated macrophages have the M1 phenotype and those exposed to living HD have M2. Stimulation with LPS enhanced the expression of *CCL1* and *CCL22* in ESP/M and reduced *CCL1* in HD/M, whereas *CCL22* was highly induced in the HD/M system. CCL1 is known as an essential chemokine for the maintenance of M2b macrophage properties. CCL22 is a M2 phenotype marker, and together with CCL1, is a Treg-attracting chemokine (44, 45).

The reduced levels of IL-1β, TNF-α, IL-6, IL-23, and IL-12p70 indicate that ESP has superior immunosuppressive properties compared to the whole parasite. Although qPCR analysis confirms that ESP inhibits IL-1β and TNF-α, the ELISA results indicate the opposite for HD stimulation without LPS, where qPCR data indicated higher TNF-α expression. However, it is known that high levels of mRNA expression do not necessarily reflect the amount of protein in a cell. Regulation of gene expression at transcriptional and translational levels (such as alternative splicing, RNA stability influenced by regulatory elements, different half-lifes of the proteins during different conditions or modification) can lead to a weak correlation between mRNA and protein levels. Despite the progress in methodology, still little is known about specificity of translation regulation, feedback and coupling between regulatory, the roles of miRNAs, RNA-binding proteins, and yet unknown mechanisms of protein abundance regulation (36). For instance, microRNAs can simultaneously downregulate hundreds of genes by inhibiting mRNA translation into protein and thus modulating many cellular processes (46).

Discrepancies are also present regarding the levels of IL-10 and IL-12; the ELISA test examined the p70 subunit, containing p40 and p35, while qPRC examined the p35 subunit. While qPCR indicated greater expression of the IL-12 p35 subunit in all cases, ELISA analysis revealed inhibition of IL-12 p70. The IL-12 is composed of the p35 (encoded by *Il12a*) and p40 (encoded by *Il12b*) chains and principally activates NK cells and induces CD4^+^ T lymphocytes to become IFN-γ-producing Th1 cells (47). The p40 chain can also form a dimer with p19 to give rise to IL-23, which is required for Th17 differentiation (48, 49). Similarly, the p35 chain can combine with Epstein–Barr-induced 3 (EBI3) to form IL-35 in induced regulatory T cells (iTr35) and tolerogenic human DCs (50). *T. muris* infection has been shown to induce the expansion of suppressive IL-35-producing CD4^+^ Foxp3^−^ “Tr35” cells in the murine intestine (51). Our analysis identified high expression of the *IL-12p35* subunit and ELISA validation revealed inhibition of IL-12p70 and IL-23, which may suggest that high expression of p35 is connected with the induction of IL-35, however, this has to be elucidated in future experiments.

Another effect of HD ESP is diminished expression of ICAM-1 (CD54), a transmembrane protein expressed on epithelial cells, endothelial cells, and immune cells such as T cells and macrophages. ICAM-1 enables leukocytes to migrate through the endothelia to the inflammation site (52), participates in immunological synapse formation (53), and it is also implicated in the formation and progression of atherosclerotic lesions (54) and development of experimentally induced intestinal inflammation (55). Interestingly, our study revealed an increased level of sICAM-1 in culture media from ESP-treated cells. This supports the findings of previous studies which suggest that ICAM-1 is not only expressed on the cell membrane (mICAM-1), but is also released as a soluble molecule (sICAM-1), possibly resulting from proteolytic cleavage or alternative RNA splicing (56, 57). This may explain the difference in expression levels of mRNA and protein, especially when there are reports about the presence of separate distinct messenger RNA transcripts coding for mICAM-1 and sICAM-1 (58). The binding of sICAM-1 to LFA-1 is capable of inhibiting lymphocyte attachment to endothelial cells (59); however, the role and functions of soluble ICAM-1 have not yet been completely elucidated. Some analyses have revealed that sICAM-1 plays a role in neutrophil inhibition and macrophage recruitment during inflammation. sICAM-1 can act as a regulator during inflammatory processes. Excessive circulating sICAM-1 in transgenic animals may bind to β2 integrin on the leukocyte and thus decrease its availability for cell–cell interactions (60). All of the above could represent a parasite immune evasion strategy aimed to block leukocyte:endothelial cells interactions.

Recent experiments show that helminth derived molecules may reduce lupus-associated accelerated atherosclerosis in a mouse model (61) and offer strong protection against cholesterol-induced atherosclerosis development (62). Our data show a correlation between the presence of parasite and expression of a macrophage CD36 receptor. This macrophage scavenger receptor is responsible for recognition and internalization of oxidized lipids, and represents a major participant in atherosclerotic foam cell formation (63). In addition, human studies have shown CD36 to be associated with impaired insulin sensitivity (64–66) and pathogenesis of metabolic disorders such as insulin resistance, obesity, and non-alcoholic hepatic steatosis, and an absence of CD36-mediated lipid uptake in muscle or liver is capable of preventing diet-induced lipotoxicity (67–69). Our results show evident reduction in the expression of CD36 in cells stimulated with ESP and HD. For those stimulated with ESP, downregulation was also observed in the presence of LPS. This may indicate one of the mechanisms used by parasites for immunomodulation and could explain the beneficial effects of parasites on atherosclerosis and metabolic disorders.

As HD ESP demonstrated greater downregulation of gene expression for inflammatory cytokines and chemokines, it was decided to analyze the phosporylation profiles of a range of signaling proteins from various pathways.

Changes in phosphorylation profiles were observed after stimulation with ESP and HD. The phosphorylation level of the following proteins was elevated after HD ESP treatment, irrespective of LPS application: ERK 1/2, Akt 1/2/3 T308, p53 S392, p53 S46, and HSP 60. Studies concerning the lacto-*N*-fucopentaose III (LNFPIII) carbohydrate moiety present on *S. mansoni* eggs and ES-62 reveal that sustained ERK activation can suppress Th1-inducing IL-12 production (70). Inhibition of the production of the shared p40 subunit, mediated by ERK, can also cause downregulation of IL-12p70 and IL-23 cytokines, as pretreatment of cells with an ERK pathway inhibitor can reduce IL-12p40 production (71–73).

Another important molecule is β-catenin, a ubiquitously expressed main signal transducer of the canonical Wnt signaling pathway. Activation of the Wnt/β-catenin pathway with Wnt3a in mouse microglial cells leads to the expression and release of the pro-inflammatory cytokines interleukins IL-6, IL-12, and IFNγ (74). In contrast, the Wnt/β-catenin pathway has also been demonstrated to play an anti-inflammatory role in mouse colon epithelial stem cells and macrophages infected with *Salmonella* (75) or *Mycobacterium* (76), which indicates that activation of the Wnt/β-catenin pathway downregulates the pro-inflammatory responses to certain bacterial infections (77, 78). Our results show that β-catenin has a higher phosphorylation level in ESP-stimulated cells, which could contribute to the immunosuppressive properties of these proteins.

Significantly greater induction of phosphorylation was observed in c-JUN, HSP27, and CREB after stimulation with HD compared to ESP. Several studies attribute an anti-inflammatory function to HSP27. Stimulation of THP-1 macrophages with recombinant HSP27 resulted in increased NF-κB transcriptional activity and induced the expression of a variety of genes, including the pro-inflammatory factors IL-1β and TNF-α. However, it was also found to increase the expression of anti-inflammatory factors including IL-10 and GM-CSF (79). Our results reveal greater expression of IL-1β at both the gene and protein levels, and increased expression of *TNF-*α and *IL-10* at the gene level following stimulation with HD and LPS. Induction of the inflammatory cytokine might be via the JNK pathway, where c-Jun is a component of the AP-1 transcription factor. This pathway appears to play a significant role in chronic inflammatory diseases involving the expression of specific proteases and cytokines (80, 81); however, the expression of pro-inflammatory cytokines is independent of the JNK/AP-1 signaling cascade in human neutrophils (82). In addition to these findings, HD incubation with cells induces transcription factor CREB, which is known for its role in cell proliferation, differentiation, and survival (83–85). However, recent evidence has revealed its function in immune responses, including inhibiting NF-κB activation, inducing macrophage survival and promoting the proliferation, survival, and regulation of T and B lymphocytes. While some studies identify CREB as a part of the anti-inflammatory immune response (86), others associate it with the pro-inflammatory response (87). As the qPCR analysis revealed upregulation of inflammatory cytokine and chemokine expression in the case of HD treatment, our present results favor a pro-inflammatory response.

STAT2, AMPKα1, and Akt 1/2/3 S473 demonstrated greater phosphorylation following ESP + LPS compared to HD + LPS. While STAT2 may be a novel regulator in the immunosuppressive function of mesenchymal stem cells (88), AMPKα1 is crucial for phagocytosis-induced macrophage skewing from a pro- to anti-inflammatory phenotype at the time of resolution of inflammation (89). Akt is a major metabolic regulator implicated in M2 activation (90, 91). It mediates enhanced glucose consumption in M2 macrophages, which contributes to induction of M2 gene expression (92). These results indicate the induction of an M2 phenotype in the presence of ESP and LPS.

## Conclusion

We examined several proteins/kinases, which have never been considered in molecular studies devoted to host–parasite interactions, and which are now intended for more in-depth study in subsequent experiments to assess their relevance in the immune response. The information revealed in this report may allow for the discovery of new signaling pathways, or improve our understanding of those already identified. With knowledge of the beneficial effects of parasites on many immune related diseases, such research can contribute to a better understanding of these diseases and the mechanisms underlying them. As shown above, our results suggest the presence of markers for both M1 and M2 macrophage phenotypes; therefore, we can conclude that infections with adult tapeworms induce mixed polarization of macrophages. This may explain the phenomenon where adult cestodes, although attached to the host intestinal epithelium with their adhesive structures and billions of microtriches covering the surface layer of the parasite tegument do not harm the hosts and usually do not induce an inflammatory reaction. We hope our results pave the way for future in-depth studies to find and elucidate novel mechanisms involved in parasite immunomodulation, especially regarding helminth infections that are known to have a considerable influence on a number of serious autoimmune diseases, in which a careful experimental approach is necessary. Bearing in mind that our previous report confirmed that HD ES products have immunogenic properties (18), our next step will be focused on the characteristics and careful analysis of immunomodulatory functions of single identified proteins. This may be essential to establish the molecules involved in the mechanisms of immunomodulation and determine the mechanism of their action.

## Author Contributions

AZ-D, KB, BS, and DM performed the experiments; designed the experiments; interpreted the data; drafted the manuscript; reviewed and approved the final version of the manuscript; agreed to be accountable for the content of the work.

## Conflict of Interest Statement

The authors declare that the research was conducted in the absence of any commercial or financial relationships that could be construed as a potential conflict of interest.
