# Supplementary material for: New Data on Human Macrophages Polarization by Hymenolepis diminuta Tapeworm—An In Vitro Study
Source: Front Immunol. 2017 Feb 20;8:148. doi: 10.3389/fimmu.2017.00148 (PMC5316519; doi:10.3389/fimmu.2017.00148)
Supplement: Supplementary file 1 [file Data_Sheet_1.docx]

Supplementary Material

**New data on human macrophages polarization by *Hymenolepis* *diminuta* tapeworm – an *in vitro* study**

Anna Zawistowska-Deniziak^1*^, Katarzyna Basałaj^1^, Barbara Strojny^2^, Daniel Młocicki^1,3^

*** Correspondence:** Anna Zawistowska-Deniziak anna.zawistowska@twarda.pan.pl

# Supplementary Figures

**
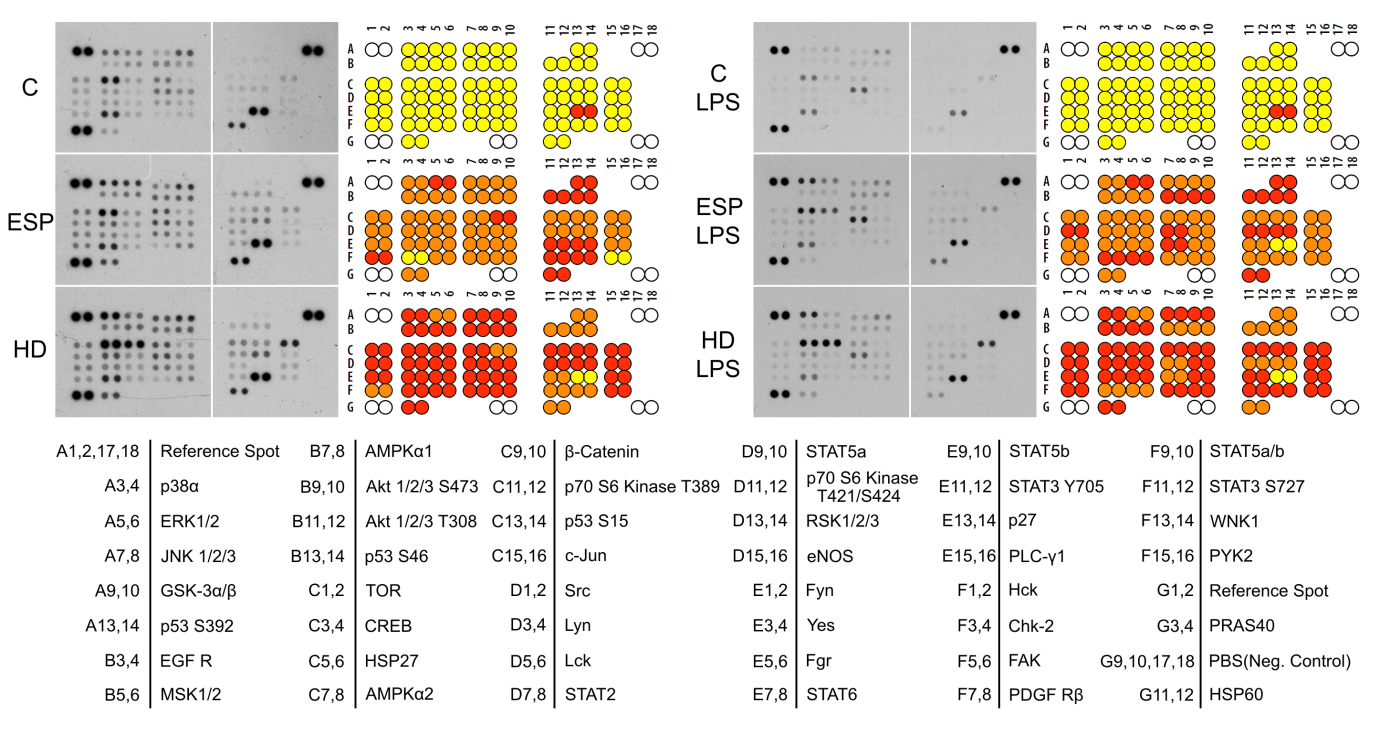
**

**Supplementary Figure 1.** **Signaling proteins phosphorylation profile.** Changes in signaling proteins phosphorylation profile in macrophages stimulated with ESP, HD, ESP+LPS and HD+LPS was determined by Proteome Profiler Human Phospho-Kinase Array Kit - a membrane-based sandwich immunoassay. Capture and control antibodies have been spotted in duplicate on nitrocellulose membranes. Cell lysates are diluted and incubated overnight with the Human Phospho-Kinase Array. The array is washed to remove unbound proteins followed by incubation with a cocktail of biotinylated detection antibodies. Streptavidin-HRP and chemiluminescent detection reagents are applied and a signal is produced at each capture spot corresponding to the amount of phosphorylated protein bound. It detects phosphorylation of 43 human kinases and total amounts of 2 related proteins simultaneously. Stimulated cells were lysed with usage of special kit buffer and frozen in -80° C until use. Protein concentration was checked and the same amount (400 µg – ESP, HD, C; 240 µg-ESP+LPS, HD+LPS, C+LPS) was used for each analysis. Analysis of control and treated sample has to be performer at one time. Dots marked with yellow – lowest, orange – higher, red- the highest level of phosphorylation.
